# Combined effect of heart rate responses and the anti-G straining manoeuvre effectiveness on G tolerance in a human centrifuge

**DOI:** 10.1038/s41598-020-78687-3

**Published:** 2020-12-10

**Authors:** Min-Yu Tu, Hsin Chu, You-Jin Lin, Kwo-Tsao Chiang, Chuan-Mu Chen, Hsin-Hui Chen, Chen-Shu Yang, Chung-Yu Lai

**Affiliations:** 1 Aviation Physiology Research Laboratory, Kaohsiung Armed Forces General Hospital Gangshan Branch, Kaohsiung City, Taiwan; 2 Department of Health Business Administration, Meiho University, Pingtung County, Taiwan; 3 Department of Life Sciences and PhD Program in Translational Medicine, National Chung Hsing University, Taichung City, Taiwan; 4 Institute of Medical Science and Technology, National Sun Yat-Sen University, Kaohsiung City, Taiwan; 5 Graduate Institute of Aerospace and Undersea Medicine, National Defense Medical Center, Rm. 8347, No. 161, Sec. 6, Minquan E. Rd., Neihu Dist., Taipei City, 11490 Taiwan; 6 Civil Aviation Medical Center, Taipei City, Taiwan; 7 The iEGG and Animal Biotechnology Center, National Chung Hsing University, Taichung City, Taiwan; 8 Department of General Medicine, Tri-Service General Hospital, National Defense Medical Center, Taipei City, Taiwan

**Keywords:** Physiology, Cardiology, Neurology

## Abstract

Increased heart rate (HR) is a reaction to head-to-toe gravito-inertial (G) force. The anti-G straining manoeuvre (AGSM) is the crucial technique for withstanding a high-G load. Previous studies reported the main effects of HR only or AGSM only on G tolerance. We assessed the combined effect of HR and AGSM on the outcome of 9G profile exposure. A total of 530 attempts for the 9G profile were extracted to clarify the association of interest. Subjects with an AGSM effectiveness of less than 2.5G had a 2.14-fold higher likelihood of failing in the 9G profile. Trainees with HR increases of less than 20% in the first five seconds also had higher odds of 9G profile intolerance (adjusted OR 1.83, 95% CI 1.09–3.07). The adjusted OR of 9G profile disqualification was 2.93 (95% CI 1.19–7.20) for participants with smaller HR increases and lower AGSM effectiveness. The negative effect of a smaller HR increase on the outcome was likely to be affected by improved AGSM effectiveness (adjusted OR 1.26, 95% CI 0.65–2.42). We speculate that low AGSM effectiveness and a small HR increase were separately associated with failure of high-G challenge. Nonetheless, good AGSM performance seemed to reduce the negative effect of weak HR responses on the dependent variable.

## Introduction

Military pilots who fly high-performance aircrafts are frequently exposed to large head-to-toe gravito-inertial (G) forces. Orthostatic stress induced by G force decreases the mean arterial pressure and blood flow velocity, leading to blood being retained in the lower extremities^1^. Pilots can develop visual disturbances, low cerebral oxygen saturation and, if without proper protection, G-induced loss of consciousness (GLOC). Several studies have indicated that the majority of military aircrew have experienced visual disturbances^2,3^ and that approximately 10–20% of them have suffered GLOC episodes in flight^4,5^.

Baroreflex, a cardiovascular response, will be fully activated to restore blood pressure and to enhance G tolerance when aircrews are subjected to sustained G stress. The average of relaxed G tolerance (RGT) is from 4.5 to 6G, determined at a gradual onset rate (GOR) run. The protective mechanism is modulated by sympathetic vasoconstriction and parasympathetic heart rate (HR) increase. HR increase could be an indicator of baroreflex activation to compensate for the drop of cerebral blood perfusion during the G exposure^6–8^. Compared with the low-G tolerance group, there were higher HR responses in the high-G tolerance group under a mild hypergravity environment^9^.

In addition to HR increase, the anti-G straining manoeuvre (AGSM) is the best countermeasure to establish pilots’ G tolerance and was developed to prevent GLOC in modern fighters. Sevilla et al*.* reported that 72% of 74 GLOC mishaps were directly related to poor AGSM performance^10^. When properly executed, the AGSM can increase an individual’s tolerance to approximately 4G^11^. High-G training with a human centrifuge is widely recognized as an effective and safe way to examine aircrew’s AGSM techniques. AGSM consists of two components: forced respiration (also called Valsalva manoeuvre) and lower body muscle strain. In the respiratory component, pilots take a preparatory breath to inflate the lung and forcefully exhale against the glottis to increase intra-thoracic pressure. Then, they make a rapid air exchange every three seconds to provide oxygenation. Simultaneously, aircrew execute an inward squeeze of lower body muscles to prevent blood pooling in the lower extremities.

Blood pressure and cerebral blood flow significantly increase during the AGSM^12,13^. HR also obviously increases with the Valsalva manoeuvre and leg contractions^14,15^. Previous studies reported the main effects of HR only or AGSM only on G tolerance. In many countries, real-time HR monitoring is performed by aviation physiologists during high-G training. Little is known about the combined effect of HR responses and AGSM effectiveness on G tolerance. A few studies have also been conducted to assess an aircrew’s tolerance under an extreme high-G load with a very high onset rate (VHOG) run in the human centrifuge for fighter pilots. Hence, the main purposes of this study were to (1) test the hypothesis that lower HR increase could be an early indicator of failed 9G exposure sustained for 15 s with a VHOG run (the so-called 9G profile) and that the effect of lower HR change would be compensated by good AGSM practice, and (2) identify possible biological variables associated with the disqualification rate of the 9G profile for the target population of fighter pilots.

## Results

During the 8-year study survey, we extracted data on 530 attempts for the 9G profile from training data records. There were 428 (80.76%) attempts by subjects who completed the profile (qualified group) and 102 (19.24%) attempts by subjects who did not tolerate the profile for 15 s (disqualified group). In the disqualified group, in 7 (6.9%) attempts, the G load was terminated by the trainee themselves. Compared with the disqualified group, as shown in Table 1, the qualified group was more likely to be younger and to have a body mass index (BMI) higher than 21 kg/m^2^. RGT, straining G tolerance (SGT), and AGSM effectiveness in the qualified group were also significantly higher than those in the disqualified group. As shown in Table 2, the majority (71.57%) of failed attempts occurred during the initial 1–5 s. In nearly one-fourth of the attempts, subjects had the ability to tolerate the profile for 6–10 s; in fewer than 5% of the attempts, subjects could stay in the 9G environment for more than 10 s.

**Table 1:** Characteristics of the 9G profile training attempts.

Variables	Qualified (N = 428)	Disqualified (N = 102)	*P* value
Age (years)	25.35 ± 0.97	26.40 ± 1.74	< 0.001
Height (cm)	173.75 ± 5.19	174.64 ± 5.53	0.125
Weight (kg)	72.76 ± 9.17	72.37 ± 10.23	0.702
**BMI (kg/m**^**2**^**); N (%)**			< 0.001
≥ 21	375 (87.62%)	78 (76.48%)	
< 21	53 (12.38%)	24 (23.52%)	
**RGT (G); N (%)**			< 0.001
≥ 5	304 (71.03%)	41 (40.20%)	
< 5	124 (28.97%)	61 (59.80%)	
**SGT (G); N (%)**			< 0.001
≥ 8	316 (73.83%)	18 (17.65%)	
< 8	112 (26.17%)	84 (82.35%)	
**AGSM effectiveness (G); N (%)**			< 0.001
≥ 2.5	303 (70.79%)	52 (50.98%)	
< 2.5	125 (29.21%)	50 (49.02%)	

*N* number, *BMI* body mass index, *RGT* relaxed G tolerance, *SGT* straining G tolerance, *AGSM* anti-G straining manoeuvre.

**Table 2:** Distribution of the duration for which individual sustained 9G exposure.

Variables	Qualified (N = 428)	Disqualified (N = 102)
1–5 s; N (%)	0 (0.00%)	73 (71.57%)
6–10 s; N (%)	0 (0.00%)	25 (24.51%)
11–14 s; N (%)	0 (0.00%)	4 (3.92%)
15 s; N (%)	428 (100.00%)	0 (0.00%)

*N* number.

Although the HRs at baseline and before the GOR test were not substantially different between the two groups, the HR before the 9G load in the disqualified group was significantly higher than that in the qualified group [145.18 ± 16.89 beats per minute (bpm) vs. 140.48 ± 18.41 bpm, *P* value = 0.019)]. However, there were no differences in the mean maximal HR in different phases of 9G exposure between the study groups. We calculated the HR increase ratio by dividing the maximal HR by the HR prior to 9G profile exposure and found that the ratio from 1–5 s was obviously lower among the disqualified trainees than among the qualified trainees (disqualified vs. qualified: 1.16 ± 0.15 vs. 1.22 ± 0.20, *P* value < 0.001). HR increased by more than 20% for 40% of the qualified attempts, but only 27.5% of the disqualified attempts achieved this level (*P* value = 0.013), as presented in Table 3.

**Table 3:** HR changes at different stages of training.

Variables	Qualified (N = 428)	Disqualified (N = 102)	*P* value
HR at baseline (bpm)	109.32 ± 16.17	112.04 ± 15.45	0.125
HR before GOR test (bpm)	122.70 ± 16.92	125.58 ± 15.65	0.118
HR before the 9G profile (bpm)	140.48 ± 18.41	145.18 ± 16.89	0.019
**Maximal HR during the 9G profile (bpm)**			
1–5 s	169.07 ± 22.56	167.06 ± 21.54	0.414
6–10 s	175.81 ± 18.59	179.45 ± 22.02	0.314
11–15 s	179.61 ± 18.57	189.50 ± 16.78	0.289
HR increase ratio*			
1–5 s	1.22 ± 0.20	1.16 ± 0.15	< 0.001
6–10 s	1.27 ± 0.20	1.22 ± 0.16	0.193
11–15 s	1.30 ± 0.19	1.20 ± 0.09	0.212
**HR increase; N (%)**			
≥ 20%	175 (40.89%)	28 (27.45%)	0.013
< 20%	253 (59.11%)	74 (72.55%)	

*N* number, *HR* heart rate, *GOR* gradual onset rate.

*Maximal HR during the initial 1–5 s of the 9G profile divided by HR before the 9G profile.

As illustrated in Table 4, multivariate logistic regression indicated an elevated adjusted odds ratio (OR) for an older age [OR 1.99; 95% confidence interval (CI) 1.61–2.46] as well as a BMI of less than 21 kg/m^2^ (OR 2.02; 95% CI 1.12–3.64). As expected, AGSM effectiveness in the GOR profile was a predictor of the outcome of 9G profile training. Subjects with an AGSM effectiveness of less than 2.5G had a 2.14-fold higher likelihood of failing in the 9G profile than did those with an AGSM effectiveness of more than 2.5G. In addition, trainees with an increase in HR of less than 20% from 1–5 s also had an increased odds of 9G profile intolerance (OR 1.83; 95% CI 1.09–3.07).

**Table 4:** Multivariate analysis of 9G profile training tolerance performed with logistic regression.

Variables	Qualified (N = 428)	Disqualified (N = 102)	Adjusted OR (95% CI)	*P* value
Age (year)	25.35 ± 0.97	26.40 ± 1.74	1.99 (1.61–2.46)	< 0.001
**BMI (kg/m**^**2**^**); n (%)**				
≥ 21	375 (87.62%)	78 (76.48%)	Ref	
< 21	53 (12.38%)	24 (23.52%)	2.02 (1.12–3.64)	0.020
**AGSM effectiveness (G); N (%)**				
≥ 2.5	303 (70.79%)	52 (50.98%)	Ref	
< 2.5	125 (29.21%)	50 (49.02%)	2.14 (1.33–3.45)	0.002
**HR increase ratio**^**✽**^**; N (%)**				
≥ 20%	175 (40.89%)	28 (27.45%)	Ref	
< 20%	253 (59.11%)	74 (72.55%)	1.83 (1.09–3.07)	0.023

*N* number, *BMI* body mass index, *AGSM* anti-G straining manoeuvre, *HR* heart rate, *OR* odds ratio, *CI* confidence interval.

*Maximal HR during the initial 1–5 s of the 9G profile divided by HR before the 9G profile.

Table 5 further describes the impact of the HR increase ratio on the dependent outcome stratified by AGSM effectiveness after adjustments for the confounder. In the group with an AGSM effectiveness of less than 2.5G, participants with an HR increase of less than 20% would have a 2.93-fold increased likelihood of failure in the 9G profile. Nevertheless, the negative effect of inadequate HR increase on the training outcome was likely to be neutralized among those participants with an AGSM effectiveness of more than 2.5G (OR 1.26; 95% CI 0.65–2.42).

**Table 5:** Combined effect of HR increases and AGSM effectiveness.

Variables	AGSM effectiveness < 2.5G		AGSM effectiveness ≥ 2.5G	
	Adjusted OR^†^ (95% CI)	*P* value	Adjusted OR^†^ (95% CI)	*P* value
HR increase ratio*				
≥ 20%	Ref		Ref	
< 20%	2.93 (1.19–7.20)	0.019	1.26 (0.65–2.42)	0.493

*HR* heart rate, *BMI* body mass index, *AGSM* anti-G straining manoeuvre, *OR* odds ratio, *CI* confidence interval.

^†^Model adjusted for age, BMI.

*Maximal HR during the initial 1–5 s of the 9G profile divided by HR before 9G profile.

## Discussion

Using this valuable database, we investigated the effects of HR changes and AGSM effectiveness and assessed the determinants of the dependent variable. The results showed that older age, a lower BMI, poor AGSM effectiveness and a smaller increase in HR can increase the likelihood of failure during high-G training. AGSM effectiveness seemed to influence the negative effect of a smaller increase in HR on the training outcome among these fighter pilots.

G force decreases individuals’ arterial blood pressure and impedes blood perfusion to the brain. Blood redistribution is sensed by aortic and carotid baroreceptors and activates cardiovascular responses such as increases in HR, cardiac contractility, and peripheral resistance. The data from previous studies have demonstrated that HR elevates by 10 bpm for each G increment in the steady state conditions during gradual acceleration^16^. With exposure to high-G stress under a VHOG run, the non-GLOC subjects had a larger increase in HR than did the GLOC subjects during training^17^. Overall, HR changes can not only play an important role in G tolerance but also be indicative of the regulation of baroreflex.

In the central nervous system, a small amount of oxygen or energy reserve is metabolized and released under hypoxic conditions. The residual oxygen helps trainees maintain normal function for approximately 5 s under rapid onset and sustained high-G load^18^. GLOC also frequently occurred after 5 s of exposure to G force in a VHOG run in our study^19^. We additionally recorded trainees’ maximal HR at the plateau of 9G every 5 s. The findings showed that the qualified group had an obviously higher percentage of HR elevation, although the maximal HRs during the first phase did not differ between the two groups. This result may have been because the HR before the 9G profile was higher among disqualified trainees than among qualified trainees. In other words, the results suggested that disqualified subjects had a lower ability to recover from the prior profiles.

A faster HR recovery after short-interval and high-intensity training also indicated optimal physical fitness and performance^20–22^. For the daily physical training, a well-designed programme to enhance fighter pilots’ G tolerance should put the balance between strength training and aerobic training into consideration. Moderate amounts of aerobic training would strengthen cardiovascular function and shorten the recovery time from the repetitive G exposures. Disqualified trainees were recommended to keep improving their physical conditions before the next attempt. We further analysed the HR response data among the 25 qualified subjects who undertook more than two attempts. The average HR before the 9G profile in disqualified attempts was higher than in the qualified attempts, reaching borderline significance (disqualified vs. qualified: 148.76 ± 18.00 bpm vs. 141.72 ± 18.90 bpm, *P* value = 0.054). This discovery might also reflect the important role of recovery level on G tolerance.

In addition, subjects with a higher HR reserve, the difference between the maximum and minimum resting heart rate, also presented a greater ability to recover from the orthostatic stress created by the G force^9,23,24^. Our work illustrated that the maximal HRs in the initial 5 s of the 9G profile were not different between the disqualified and qualified groups. However, the HR before the 9G profile was higher among the disqualified trainees, indirectly suggesting the different levels of HR reserve between the two groups. Because HR reserve was not evaluated in the current study, the phenomenon observed will be examined to determine whether the pass rate is related to the level of HR reserve in another work.

The AGSM is known to be the most effective method to prevent GLOC^25,26^. Similar to previous reports, trainees in our study had average RGT that ranged from 4.5 to 6.0G, and more than 80% of these subjects’ SGT was above 8G^27^. In this study, AGSM effectiveness was calculated by subtracting the G level at SGT from the G level at RGT during the GOR profile and was expressed in G units. The findings not only revealed that RGT and SGT were positively correlated with the qualified rate in the 9G profile but also showed that AGSM effectiveness was related to G tolerance^27,28^. We found that AGSM effectiveness was only moderately associated with G tolerance during training. The three potential explanations are as follows: (1) there was within-subject variability in the subjective vision loss results used to determine whether subjects met the vision loss criteria during the test^29^; (2) the centrifuge was designed to automatically decelerate when the G force reached the 9G upper limit in the GOR profile; and (3) trainees terminated the AGSM early and conserved their energy for the next three upcoming VHOG profiles. To minimize inter-subject measurement error and to improve the reliability of the results in the study, a larger sample was chosen. However, the association between AGSM effectiveness and the 9G profile qualification rate was still underestimated in this study.

In our multivariate model, a lower proportion of HR responses had an adverse impact on pass rate, but better AGSM effectiveness corresponded to an increased likelihood of 9G profile qualification. We further extended the analysis by performing stratification. The observations indicated that the effect of a smaller HR increase ratio associated with the failure rate seemed to be affected by the AGSM effectiveness variable. Effective AGSM performance could strongly neutralize the detrimental effect of a small cardiovascular reaction. Possible explanations are as follows: First, van Lieshout et al*.* revealed that HR and blood pressure increase considerably after the Valsalva manoeuvre^14^. Good performance of the respiratory component of the AGSM can augment cardiovascular function during high-G exposure. Second, the isometric straining of lower body muscles during the AGSM increases peripheral resistance and venous return to the heart^12,30^. Therefore, good AGSM operation could alleviate the G stress to maintain the volume of blood supply. Finally, when performed well, the AGSM establishes sufficient cardiovascular tolerance against G stress. Simultaneously, it might have improved the balance between sympathetic and parasympathetic tone and reduced the negative effects of insufficient HR increase ratio.

According to the literature review, some biological factors might be associated with G tolerance in flight and during human centrifuge training. In the 1980s, Lyons et al. stated that GLOC mishap pilots had significantly less aircraft-specific flight time than did other pilots^26^. Sevilla et al*.* indicated that 32% of cases in F-15, F-16, and A-10 aircraft occurred in student pilots^10^. Green et al*.* found that over 50% of pilots with GLOC experience had a small number of total flight hours^31^. Pilots with a younger age and less aircraft-specific or total flight time were more likely to experience in-flight GLOC. In centrifuge training, age and years of flight experience are not related to GLOC among well-experienced aviators^32^. Before beginning the official pilot training course, senior trainees had a higher likelihood of passing the centrifuge test than did beginners^28^. It seemed that age and years of flight experience might be associated with pilot performance during high-G training. However, in contrast with the conclusion mentioned above, our results showed that the disqualified group was older than the qualified group. The reason for this difference could be that in our study, trainees were allowed several attempts to pass the 9G profile in advanced high-G training. The next attempt was scheduled after a specific time period following the previous failed attempt. Therefore, trainees are older during their next attempt to pass training. The hours of flight factor was not an important issue in the current study because all subjects underwent the same flying training course and had approximately 250 flight hours.

Corresponding to previous results, there was no relationship between BMI, analysed as a continuous variable, and the pass rate in the centrifuge^32,33^. In the present study, we additionally categorized trainees into high and low BMI groups and discovered that there was a higher disqualified rate in the low BMI group. For high-G aircrew, physical conditioning is an effective method to enhance the G tolerance. The main training component focuses on anaerobic programmes, which will potentially increase muscle mass and lead to higher BMIs among those participants with good physical fitness. In the future, we will further assess the partial effect of BMI or body composition on G tolerance.

This study was designed to highlight the interactive impact of HR responses and AGSM proficiency on the outcomes of fighter pilots undergoing high-G training, which has rarely been investigated in the past. We also elucidated the parameters associated with G tolerance in our country. This study had some inherent limitations. First, the subjects were not naïve high-G trainees, as they had completed an intermediate course. They had learned the fundamentals of the AGSM technique and underwent physical adaption. Thus, it is expected that the association of interest was underestimated. Second, we did not have any information on fatigue or physical conditions related to AGSM effectiveness^34,35^. The influence of residual confounders could not be excluded from the present study. Third, because G tolerance was determined by a complicated mechanism, important cardiovascular variables related to G tolerance were not recruited in this study due to the data limitations. We must conservatively use the HR responses as a preliminary indicator to assess the outcome of high-G training. Next, due to technical limitations, blood pressure was not measured in the centrifuge training. Thus, we could not investigate the causal relationship between the outcome and change of blood pressure. In the future studies, blood pressure will be recorded during the high-G training to strengthen the association between G tolerance and haemodynamic variables. Finally, all trainees wore the inflated anti-G suit during the 9G profile in accordance with regulations. We could not eliminate the effect of the anti-G suit, which might reduce the strength of the conclusions.

In conclusion, individuals with older age, a lower BMI, and less RGT, SGT and AGSM effectiveness may be less likely to qualify for the 9G profile. Subjects with smaller HR increases or those who had less AGSM effectiveness were more likely to be disqualified. The findings in the univariate analysis were consistent with those in the multivariate analysis. According to the stratification results, good AGSM effectiveness seems to relieve the influence of a smaller HR increase ratio on the main outcome.

## Method

### Study design and data sources

We conducted a retrospective study using the high-G training worksheet and electronic data retrieved from the Aviation Physiology Research Laboratory (APRL). The APRL is in charge of aviation physiology and high-G training for military aircrew in Taiwan. Approximately three hundred trainees attended acceleration training with a human centrifuge (Latécoère, Toulouse, France) each year.

Data on high-G training were also recorded by well-trained aviation physiologists. The instructors and senior aviation physiologists supervised the validity of the documents and electronic video files after each high-G training session and stored them in a restricted room. A nonspecific code was randomly assigned to each participant to protect confidentiality and privacy.

### Training procedures and eligible subjects

Subjects selected were fighter pilots who had previously completed intermediate high-G training. Qualification for the advanced high-G training course, including lectures, skill exercise and completion of four centrifuge profiles, is a prerequisite for transition to high-performance fighters.

Details of the four centrifuge profiles are as follows: (1) GOR run (0.1G/s) to examine the RGT and SGT in accordance with subjective peripheral vision loss; (2) VHOG (6G/s) run of 6G for 30 s to practice the AGSM under moderate G stress; (3) VHOG (6G/s) run of 9G for 15 s (9G profile) to confirm AGSM effectiveness under the G stress matching high-performance fighters; and (4) VHOG (6G/s) run of 7G for 10 s at the check-six position. Essentially, subjects needed to undertake and complete all four profiles sequentially in one day. They were trained in a seat reclined by 30 degrees and wore an anti-G suit inflated from profile 2 to profile 4. Between the profiles, they rested at 1.4G idle run inside the human centrifuge. We adopted profile 3 as the target profile because the goal of advanced high-G training is for subjects to sustain 9G stress for 15 s (9G profile). If subjects failed to sustain 15 s at 9G, they needed to retake the four profiles in the next attempt.

All subjects were qualified for annual check-up, and clearance was given by the squadron flight surgeon and aviation physiologist before the training. Some subjects were still excluded from the analysis if they (1) had no 9G profile attempt due to self-reported physiological discomfort; (2) were of female gender due to the small number of cases; (3) had incomplete or missing personal data; or (4) had no electrocardiogram signal on recorded video. From 2011 to 2019, data from 530 attempts from 436 subjects were extracted for statistical analysis. Most trainees (n = 404, 92.7%) passed the 9G profile on the first attempt. A total of 10 (2.3%) subjects completed the 9G profile on the second attempt, and 22 (5.0%) subjects completed the 9G profile after three attempts or more. Only 7 subjects were disqualified from 9G profile training.

### Definitions of variables

The dependent outcome analysed in this study was the proportion of failed attempts to tolerate 15 s in the 9G profile. The subjects themselves could have terminated the G load, or the instructors could have decelerated the human centrifuge because they had already experienced GLOC.

Covariates included trainee biological data and HR responses at different stages of high-G training. Regarding biological factors, age, height, weight, BMI, RGT, SGT, and AGSM effectiveness were retrieved from the training documents. RGT was defined as the G value when trainees detected the 100% loss of peripheral vision or the 50% loss of central vision under the GOR run (profile 1), at which point they started to perform the AGSM. SGT was defined as the G level when performing the AGSM at which the trainees again met the criteria of vision loss mentioned above or the 9G upper limit. Inside the centrifuge, there were light bars as a visual reference to assess the percentage of vison loss^27,36,37^. AGSM effectiveness was defined as the difference between RGT and SGT. In the VHOG runs (from profile 2 to profile 4), trainees began to perform the AGSM following the command of the aviation physiologist immediately before the onset of G exposure.

HRs of all subjects were monitored by 12-lead electrocardiogram (Infinity CentralStation MS26800, Dräger, Telford, PA, USA). Before the centrifuge started to spin, subjects sat and rested inside the centrifuge gondola for five minutes. We retrieved subjects’ resting HR from the records documented by aviation physiologists and recorded the value as the baseline. During the training, HRs were continuously monitored and stored as electronic video files separately for the four different profiles. HR at the 1.4G idle run was recorded before the GOR ride. The maximal HR was recorded before and during the 9G profile (three phases: 1–5, 6–10, and 11–15 s). The HR increase ratios in every phase were computed as the maximal HR divided by the HR before 9G.

### Data analysis

In the univariate analysis, the subject characteristics were summarized as the means, standard deviations, numbers, or percentages, as appropriate. The distributions of all the data between the qualified and disqualified groups during the 9G profile were compared by the independent samples t-test or the Mann–Whitney U test for continuous factors and the chi-square test for discrete variables. Variables that reached a significance level with a two-tailed *P* value of < 0.05 in the univariate test were included in the final model.

To investigate independent factors affecting the training outcomes, binary logistic regression with the enter model was used to estimate the OR and 95% CI. The combined effect of HR increases and AGSM effectiveness on the main outcome was assessed by stratification. A *P* value of < 0.05 for the two-tailed test was regarded as significant. All analyses were performed with SPSS 26.0 for Windows (IBM, Armonk, NY, USA).

### Ethics approval

The research method was performed in accordance with relevant regulations in Taiwan, and ethics approval was received from the Institutional Review Board of Kaohsiung Armed Forces General Hospital in Kaohsiung City, Taiwan (No. KAFGH 108-018). Because all data of this study were de-identified and anonymized, the protocol was determined to be exempt from informed consent. Reporting of the results of this study followed the STROBE guidelines.

## Data Availability

Data were obtained from the Aviation Physiology Research Laboratory, Taiwan. Due to the regulations, data used in this study are restricted and cannot be shared publicly.
